# Commentary: Immunometabolic control of cytokine production by micronutrients in health, aging, and inflammation

**DOI:** 10.3389/fimmu.2026.1904982

**Published:** 2026-07-28

**Authors:** ZhiTong Qu, WenQin Wang, Jie Wang

**Affiliations:** 1 Qingdao Municipal Hospital (Qingdao Hospital, University of Health and Rehabilitation Sciences), Qingdao, China; 2 School of Medicine, QingDao Central Hospital, University of Health and Rehabilitation Sciences, Qingdao, China

**Keywords:** CD38, NADH (NAD+), epegenetics, immunometabolic, micronutrients, clinical translation

## Introduction

In their recent publication, Ghosh and Chattopadhyay present a hierarchical immunometabolic framework with five sequential checkpoints: nutrient sensing, epigenetic programming, calcium-redox signaling, inflammasome/lytic cell death, and NAD^+^–CD38–SIRT–mediated immunosenescence (1). This innovative model admirably reframes micronutrients not merely as passive cofactors but as active metabolic sensors and epigenetic modifiers that dynamically shape immune cell fate and functional polarization across both innate and adaptive immune cell compartments. In this commentary, we discuss five key dimensions that we believe deserve further consideration to strengthen the mechanistic coherence and translational applicability of this framework: convergent hub architecture, stress-mediated neuroendocrine inputs, extended epigenetic mechanisms, a redox threshold circuit, and the critical role of intervention timing.

## Reconsidering the CD38–NAD^+^–SIRT axis as a convergent hub

While Ghosh and Chattopadhyay position the NAD^+^/CD38/SIRT axis as a fifth, parallel checkpoint (CP5), we propose that the available evidence supports reconsidering it as the convergent hub that mechanistically integrates all five checkpoints rather than operating in parallel. NAD^+^ availability governs epigenetic (SAM-dependent methylation at CP2), metabolic (mTORC1/GCN2 via SAMTOR at CP1), and redox (SIRT3/GPX4-mediated ferroptosis suppression at CP4) processes across the entire hierarchy. NAD^+^ depletion impairs SIRT1 deacetylation of NF-κB p65 and histone H3K9, sustaining constitutive pro-inflammatory cytokine expression—a hallmark of inflammaging. In COPD, lactate-induced H4K12 lactylation upregulates CD38, simultaneously disrupting calcium homeostasis (CP3) and amplifying NLRP3 inflammasome activation (CP4); CD38 blockade, p300/CBP inhibition, or NAD^+^ precursor supplementation each reverses this phenotype independently (2), demonstrating that the axis functions as a unified regulatory node rather than an isolated checkpoint. In SLE, the CD38/NAD/SIRT1/EZH2 axis impairs CD8^+^ T cell cytotoxicity through H3K27me3-mediated silencing of perforin and granzyme genes (3). These convergent effects—spanning epigenetic, metabolic, and redox domains—suggest that designating the CD38–NAD^+^–SIRT axis as the highest-priority therapeutic target and the mechanistic backbone of the checkpoint hierarchy would strengthen the explanatory power of Ghosh and Chattopadhyay’s model.

## Stress-induced metabolic rewiring

One important consideration that is not explicitly addressed in the Ghosh–Chattopadhyay framework is the role of chronic psychological stress as a pervasive upstream modifier of immune metabolism. Sun et al. (4) elucidated a brain–liver circuit whereby chronic stress activates catecholamine/ADRB2 signaling, suppressing hepatic QPRT—the rate-limiting enzyme for *de novo* NAD^+^ synthesis from the kynurenine pathway. QPRT suppression redirects kynurenine toward kynurenic acid accumulation, depleting hepatic NAD^+^ and impairing CD8^+^ T cell cytotoxicity and tumor immune surveillance. Nicotinamide supplementation restored CD8^+^ T cell surveillance and suppressed liver cancer in stressed mice. This stress-responsive neuroendocrine checkpoint intersects with CP5 (exacerbating NAD^+^ decline), CP2 (altering AhR–IL-22 signaling via kynurenine metabolites), and CP1 (disrupting mTORC1/GCN2 balance through amino acid sensing). We therefore suggest that incorporating neuroendocrine stress as an upstream modifier would meaningfully strengthen the framework’s explanatory scope, particularly given the epidemiological association between chronic psychosocial stress and accelerated immunosenescence in aging populations.

## Extended epigenetic dimensions

We note that three epigenetic mechanisms with high mechanistic relevance to the Ghosh–Chattopadhyay framework warrant explicit recognition within its epigenetic programming checkpoint (CP2). First, HDAC11 selectively brakes FoxP3^+^ Treg suppressive function; its pharmacological inhibition confers allograft tolerance (5), complementing ATRA-driven histone acetylation and identifying HDAC11 as a druggable checkpoint regulator with relevance to autoimmunity and transplantation that is not discussed in the original article. Second, zinc/copper-dependent EZH2 catalyzes H3K27me3 silencing of Foxp3 and cytotoxic effector genes, with SAM as its obligatory methyl donor—creating a micronutrient hierarchy: B12/folate sustain SAM pools, zinc/copper stabilize EZH2 catalytic activity, while vitamin D/A promote competing activating marks (H3K27ac). This hierarchy predicts that single-micronutrient supplementation will fail when upstream SAM deficiency limits all methyl-dependent processes simultaneously, a practical limitation that the original framework does not fully address. Third, SIRT1–EZH2 crosstalk links epigenetic programming (CP2) directly to the CD38–NAD^+^–SIRT hub, as demonstrated in SLE (3), where NAD^+^ depletion simultaneously reduces SIRT1 activity and disinhibits EZH2, compounding CD8^+^ T cell dysfunction through parallel epigenetic silencing of both regulatory and effector programs.

## The uPA–NADPH–PON2 circuit: a redox threshold overlooked in the framework

A further dimension that merits comment is the uPA–NADPH–PON2 redox circuit, which is not discussed by Ghosh and Chattopadhyay. uPA upregulates PON2 in macrophages through NADPH oxidase-dependent ROS generation—a homeostatic feedback loop where pro-inflammatory signaling simultaneously engages an antioxidant countermeasure (6). When uPA-driven NADPH oxidase activation exceeds PON2 and selenium-dependent thioredoxin compensatory capacity (governed by TXNRD1 at CP3), micronutrient supplementation alone is insufficient, providing mechanistic rationale for coordinated selenium/zinc supplementation with targeted pro-oxidant inhibition. We suggest that this circuit introduces a critical threshold concept that would enrich the framework’s calcium-redox checkpoint (CP3): below the tipping point, endogenous antioxidant defenses accommodate ROS and maintain redox homeostasis; above it, irreversible oxidative damage ensues despite adequate micronutrient status, necessitating pharmacological rather than purely nutritional intervention strategies.

## Intervention timing

Ghosh and Chattopadhyay’s framework is largely agnostic to intervention timing, yet recent evidence suggests that temporal context is a critical determinant of therapeutic success that the model should explicitly address. Early NMN therapy restored valvular NAD^+^ and attenuated calcification in CAVD, whereas delayed intervention after established pathology proved substantially less effective (7). This temporal dependency—where early metabolic intervention prevents irreversible tissue remodeling but late intervention cannot reverse fixed calcific damage—likely generalizes broadly across immunometabolic checkpoints. We argue that the effective intervention window closes progressively as irreversible epigenetic and metabolic reprogramming accumulates during chronic NAD^+^ depletion, with direct implications for biomarker-guided trial design: serial NAD^+^, CD38, and SAM measurements could define the transition from reversible to fixed metabolic dysfunction and thereby stratify patients most likely to benefit from targeted immunometabolic intervention.

## Clinical landscape

The clinical significance of Ghosh and Chattopadhyay’s framework is underscored by emerging translational data, and we note several recent findings that directly validate the therapeutic relevance of the checkpoint hierarchy they describe. Phase 1/2 data with low-dose oral NMN (450 mg twice daily) in steroid-refractory ITP demonstrated that 20% achieved platelet counts ≥50 × 10^9^/L and 52% sustained responses at 8 weeks (8), confirming nutritional immunometabolism as a feasible therapeutic strategy in refractory autoimmune disease. Complementary clinical evidence includes magnesium-mediated LFA-1 activation enhancing CD8^+^ T cell effector responses to immune checkpoint inhibitors in tumor models (9), vitamin D_3_ supplementation improving rituximab efficacy via NK cell ADCC augmentation (10), and personalized biomarker-guided micronutrient regimens achieving 33–46% hs-CRP reductions in inflammatory cohorts (11). Collectively, these data validate the translational viability of the multi-checkpoint framework proposed by Ghosh and Chattopadhyay while underscoring the need for combination approaches that address convergent rather than isolated nodes.

## Conclusion

In this commentary, we have discussed several considerations that we believe would strengthen the valuable framework proposed by Ghosh and Chattopadhyay. Specifically, we suggest reconsidering the CD38–NAD^+^–SIRT axis as the convergent hub integrating all five checkpoints rather than a parallel fifth checkpoint; incorporating stress-induced neuroendocrine rewiring as an upstream modifier; expanding the epigenetic landscape to include HDAC11, EZH2, and SIRT1–EZH2 crosstalk; recognizing the uPA–NADPH–PON2 circuit as a threshold mechanism within CP3; and explicitly addressing intervention timing as a critical variable for therapeutic success. Recent clinical NMN data in ITP independently validate the translational relevance of the immunometabolic checkpoint concept advanced by Ghosh and Chattopadhyay. We hope these observations stimulate further dialogue and encourage future work incorporating composite biomarker panels (NAD^+^ flux, epigenetic signatures, neuroendocrine stress markers), combinatorial multi-checkpoint intervention designs, and context-specific NAD^+^ modulation strategies that respect temporal windows of reversibility.
